# Natural Cycle Results in Lower Implantation Failure than Ovarian Stimulation in Advanced-Age Poor Responders Undergoing IVF: Fertility Outcomes from 585 Patients

**DOI:** 10.1007/s43032-020-00455-5

**Published:** 2021-01-22

**Authors:** Maria Paola De Marco, Giulia Montanari, Ilary Ruscito, Annalise Giallonardo, Filippo Maria Ubaldi, Laura Rienzi, Flavia Costanzi, Donatella Caserta, Mauro Schimberni, Matteo Schimberni

**Affiliations:** 1 Gynecology Unit, Sant’Andrea University Hospital, Department of Medical and Surgical Sciences and Translational Medicine, Medicine and Psychology Faculty, Sapienza University of Rome, Rome, Italy; 2 Clinica Valle Giulia, G.E.N.E.R.A. Centers for Reproductive Medicine, Rome, Italy

**Keywords:** Natural cycle, Poor responder, Ovarian stimulation, Bologna criteria, IVF

## Abstract

To compare pregnancy rate and implantation rate in poor responder women, aged over 40 years, who underwent natural cycle versus conventional ovarian stimulation. This is a retrospective single-center cohort study conducted at the GENERA IVF program, Rome, Italy, between September 2012 and December 2018, including only poor responder patients, according to Bologna criteria, of advanced age, who underwent IVF treatment through Natural Cycle or conventional ovarian stimulation. Between September 2012 and December 2018, 585 patients were included within the study. Two hundred thirty patients underwent natural cycle and 355 underwent conventional ovarian stimulation. In natural cycle group, both pregnancy rate per cycle (6.25 vs 12.89%, respectively, *p* = 0.0001) and pregnancy rate per patient101 with at least one embryo-transfer (18.85 vs 28.11% respectively, *p* = 0.025) resulted significant reduced. Pregnancy rate per patient managed with conventional ovarian stimulation resulted not significantly different compared with natural cycle (19.72 vs 15.65% respectively, *p* = 0.228), but embryo implantation rate was significantly higher in patients who underwent natural cycle rather than patient subjected to conventional ovarian stimulation (13 vs 8.28% respectively, *p* = 0.0468). No significant difference could be detected among the two groups in terms of abortion rate (*p* = 0.2915) or live birth pregnancy (*p* = 0.2281). Natural cycle seems to be a valid treatment in patients over 40 years and with a low ovarian reserve, as an alternative to conventional ovarian stimulation.

## Introduction

Poor ovarian response (POR) remains a challenge of scientific and clinical relevance for in vitro fertilization (IVF) treatment because of the increased failure rate and the negative influence on the patients. Although many treatments have been suggested also with a high quantity of gonadotropins, poor responder patients are refractory to stimulation protocols with very low chance of pregnancy, resulting in less than 10% for oocyte retrieval [1]. For infertile women, there is a well-known association between advanced age and IVF treatment failure [2]. Nowadays, societal changes have imposed women to achieve educational goals and established careers before programming a pregnancy. In this context the age of women seeking pregnancy has increased, and this is progressively pointing out a worldwide social problem impacting future sustainability [3].

Up to 2011, there was the lack of a universal definition of “poor responder women”: patients were identified retrospectively after the ovarian stimulation considering the number of developed follicles and/or oocytes retrieved [4–7]. The European Society of Human Reproduction and Embryology (ESHRE) in 2011 published the so-called Bologna criteria for the definition of poor ovarian responders (POR) [8]. Many studies proposed different treatment protocols to attempt for these patients, with discordant results [9]. In this scenario, the best treatment option for poor responders is still needed to be clarified. Some evidences suggested that natural cycle (NC) works at least similar to the conventional ovarian stimulation (COS), with a pregnancy rate per cycle of 6.1 and 14.9% per transfer [10]. Natural cycle is defined as IVF treatment without any gonadotrophin administration: the biological rationale for this approach, indeed, is to obtain the natural follicle recruitment and selection and subsequent in vitro fertilization and embryo culture [11].

The objective of our study was to evaluate the fertility outcomes of NC IVF compared with conventional IVF (cIVF) carried out with high-dose ovarian stimulation in a large-size cohort of advanced age Bologna criteria poor responder women.

## Materials and Methods

This was a retrospective single-center cohort study conducted at the GENERA IVF program, in Rome, Italy. Clinical and demographic data were collected from Italian Caucasian patients’ clinical chart who underwent intracytoplasmic sperm injection (ICSI) cycles between September 2012 and December 2018.

The institutional review board evaluated and approved the study.

All included patients had previously signed written informed consent regarding collection of data for research purposes. All patients had undergone a standard evaluation for infertility, including hormone test on the second day of the menstrual cycle (mainly FSH, LH, estradiol), hysterosalpingogram, hysteroscopy, complete blood tests, and semen analysis, AMH measure irrespective of the cycle day, Antral Follicle Count (AFC) on day 2–4, in a previous cycle.

### Patients’ Selection and Eligibility Criteria

Patients were selected according to the definition of poor ovarian responders by the Bologna criteria [8] and exclusively aged ≥ 40 years.

Inclusion criteria were:

- Advanced maternal age: patients over 40 years old

and at least one of the following:

- Abnormal ovarian reserve biomarker: AMH < 0.5–1.1 ng/mL; AFC < 5–7
- Previous POR: ≤ 3 oocytes with conventional stimulation
- Two episodes of POR after maximal stimulation

Exclusion criteria were:

- Body mass index (BMI) greater than 35 kg/m2
- Irregular menstrual cycles
- Previous monolateral oophorectomy
- The presence of untreated endocrine abnormalities
- The presence of comorbidities
- Patients who underwent pre-implantation genetic testing (PGT)

### Follicles Recruitment and IVF Procedure

In the cIVF group, recombinant follitropin alfa and lutropin alfa were initiated on Day 2 of cycle. The starting dose of follitropin alfa was 225 IU/day and the dose of lutropin alfa was 75 UI/daily for all patients. Endogenous LH surge was avoided by GnRh antagonist administration when the leading follicles reached 16 mm of diameters. In both groups, from the 6th day of the cycle, the patients underwent transvaginal sonography to monitor follicle size and structures within the ovary, endometrial thickness, and estradiol and progesterone dosage.

In cIVF group, when follicle size reached 18–20 mm in mean diameter and plasma estradiol value reached at least 200 pg/ml per follicle, triggering ovulation with 10,000 IU of human chorionic gonadotropin (hCG, Gonasi HP 5000; AMSA, Rome, Italy) was performed.

In NC group, no medical treatment was administrated for selection and recruitment of follicles. From the 6th day of the cycle, patients underwent transvaginal sonography to monitor follicle size and structures within the ovary, endometrial thickness, and estradiol and progesterone dosage. When follicle size reached 16 mm in mean diameter, triggering ovulation with 10,000 IU of human chorionic gonadotropin (hCG, Gonasi HP 5000; AMSA, Rome, Italy) was performed.

### Oocyte Retrieval, Laboratory Procedures, Embryo Transfer

Thirty-six hours after the injection of hCG, oocyte retrieval was performed under ultrasound control in local or general anesthesia. We performed ICSI in all cases according to previously published procedures [12], to obtain a higher fecundation rate, and to maximize the success of embryo transfer, due to the very low number of oocytes harvested in these women, and to avoid differences in the fertilization rate among patients treated with different techniques. Patients gave their informed consent accepting the possible risks occurring to offspring from ICSI. Oocytes were observed 18 h after ICSI for their pronuclei and 44 h after insemination for embryo development.

Embryos were transferred 72 h after insemination using the Sydney embryo transfer catheter (Cook Ltd., Brisbane, Queensland, Australia). All transfer procedures were performed by the same physician to avoid inter-operator variability.

All pregnancies were confirmed by a rising titer of serum b-hCG from 12 days after embryo transfer and by ultrasound demonstration of the gestation sac 4 weeks after the transfer.

The same luteal phase support was used in all cycles: 75 mg daily of intramuscular progesterone from the day of embryo transfer.

### Main Outcomes

The main outcomes were assessment of pregnancy rate per patient, per cycle, per transfer, and the embryo implantation rate.

Pregnancy was defined as the visualization of intrauterine sac with embryo with cardiac activity at the ultrasound exam.

### Statistical Analysis

Descriptive statistics were used to characterize patients’ population. The quantitative variables are expressed as the mean and categorical variables are expressed as the median or as a number (percentage). To determine whether there was a statistically significant difference between the categories of qualitative and quantitative variables, student’s *t* test was used. The Chi-square test was used to analyze the relationship between the 2 categorical data.

Parameters analyzed were the number of cycles with oocytes, number of cycles with embryos, number of embryo transfers, pregnancy rate (per patients, per cycle started and per transfer), implantation rate (number of embryos observed by ultrasound per number of embryos transferred), abortion rate, and live birth rate. All statistical analyses were performed using the Statistical Package for the Social Sciences Statistical Package (SPSS, Inc., Chicago, IL) version 25. A *p* value ≤ 0.05 was considered to be statistically significant.

## Results

**Fig. 1:**
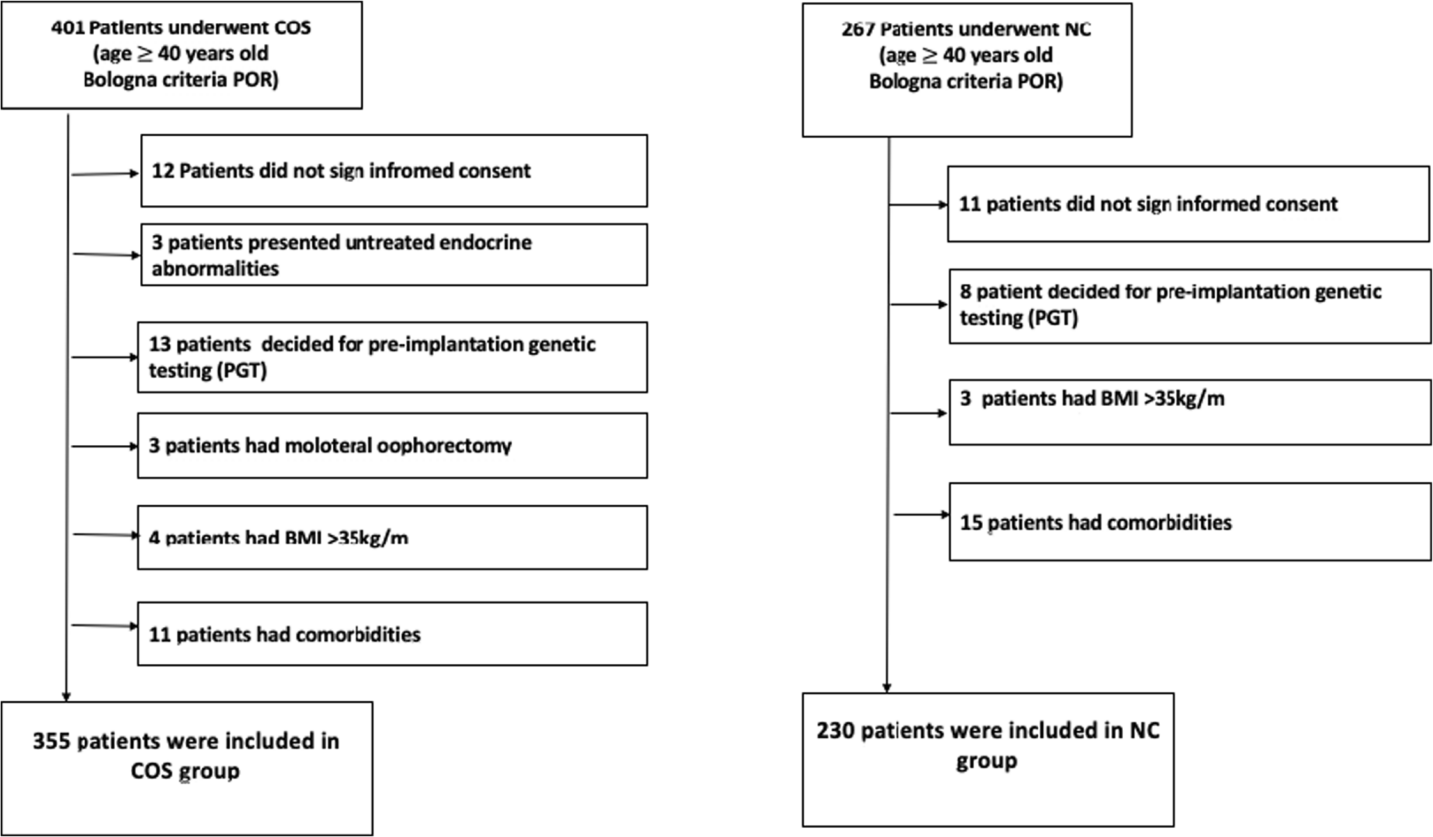
Patients selection flowchart

The patient selection flowchart is shown in Fig. 1.

Among 401 patients who underwent IVF COS protocol, 355 patients met the inclusion criteria. Among 367 patients who underwent IVF NC protocol, 230 patients met the inclusion criteria.

So 585 poor responder patients were included: 230 patients underwent NC and 355 underwent COS.

Al patients’ characteristics were listed in Table 1. Globally, the two study groups were homogeneous with regard to median age, BMI, FSH, menarche, previous pregnancies, AFC, and smoke status. AMH levels were significantly higher in stimulated patients compared with patients who underwent NC.

**Table 1:** Study group characteristics

Parameters	COS group	NC group	P value
Age (mean)	41 (40–42)	41 (40–42)	0.956
BMI	24 (22–27)	24 (22–28)	0.785
FSH	9.55	11.59	0.059
AMH	1.46	0.58	0.02
Smoke	%31,8 (113)	% 26,5 (61)	0.17
Menarche	12 (10–14)	12 (10–15)	0.654
Previous Pregnancies	4,5% (16)	2,2% (5)	0.138
AFC	4 (2–7)	3(1–5)	0.846

The total number of ovarian cycles was 1119:576 in NC patient group and 543 in COS patients group.

Reproductive outcomes are summarized in Table 2. No significant differences resulted in the number of pregnancies per patient in COS and NC groups (19.72 vs 15.65% respectively, *p* = 0.228). The abortion rate and the live birth rate were no significantly different among the two study groups. Both pregnancy rate per cycle (6.25 vs 12.89% respectively, *p* = 0.0001) and pregnancy rate per patients who underwent at least one embryo-transfer (18.85% vs 28.11% respectively, *p* = 0.025) were significantly reduced in NC group compared with COS group.

**Table 2:** Study results

Parameters	All cases	Natural cycle	Controlled ovarian stimulation	p
N. of patients	585	230	355	–
N. of cycles	1119	576	543	–
N. transfer	1042	277	765	–
Transfer/cycle	(1042/1119) 93%	(277/576) 48%	(765/543) 140%	< 0.0001
Cycles without transfer	(467/1119) 41.7%	(329/576) 57%	(138/543) 25.4%	0.00001
Pregnancy/cycle	(106/1119) 9.47%	(36/576) 6.25%	(70/543) 12.89%	0.0001
Pregnancy/ transfer	(106/1042) 10%	(36/277) 13%	(70/765) 9.1%	0.0814
Implantation rate (Pregnancy/embryos)	(106/1122) 9.45%	(36/277) 13%	(70/845) 8.28%	0.0468
Pregnancy/patients transfer	(106/440) 24%	(36/191) 18.85%	(70/249) 28.11%	0.025
Pregnancy/patients	(106/585) 18.12%	(36/230) 15.65%	(70/355) 19.72%	0.2281
live birth pregnancy	(73/106) 68.87%	(22/36) 61%	(51/70) 72%	0.2693
Abortion rate	(37/106) 34.91%	(10/36) 27.78%	(27/70) 38.57%	0.2915

The number of transfers was higher in patients who were conventionally stimulated (765 compared with 277 transfers of NC patients, *p* < 0.0001), but there was a trend towards significance for pregnancy rate per single transfer that was 13% in the NC group compared with 9.1% in COS group (*p* = 0.0814). Furthermore, embryo implantation rate resulted significantly higher in patients who underwent NC than COS (13 vs 8.28% respectively, *p* = 0.0468).

## Discussion

To our knowledge, at the present with 585 patients studied, this is the largest study in the literature with a comparison of NC, intended as a natural follicle selection and recruitment without medical treatment, with cIVF in POR patients older than 40 years. Studies on NC outcomes compared with COS in advanced age Bologna POR women are rare. Other previously published studies evaluating NC pregnancy rate examined inhomogeneous populations with no precise and standardized pre-selection of patients, with a wide diversity in the criteria used to specify POR women, thus resulting in a wide variability pregnancy rate, which ranged from 10.2 to 50% [10, 12–17]. Contrarily, our study focused specifically on the most difficult subgroup of women requiring IVF treatment, selected in accordance with the strict requirements of Bologna criteria.

Results of the present study firstly highlighted no difference in pregnancy rate per patient among women subjected to NC compared with COS.

Recently, a smaller retrospective series assessed the outcomes of the same subgroup of patients by comparing conventional stimulation with high doses of gonadotropins versus the spontaneous cycle but modified, with a minimal stimulation. It showed that the type of treatment strategy was not significantly associated with differences in ongoing pregnancy rate (OR 2.56, 95% CI: 0.9–7.6) [18].

In the present study, 15.65% of women treated with NC achieved pregnancy. These findings are more encouraging than outcomes reported in our previously published cohort, 10 years ago, when the pregnancy rate was 9.7% in poor responder patients older than 40 years managed with NC [12].

Analyzing pregnancy rate per cycle was significantly higher in cIVF than in NC. These data are in accordance with the studies that reported a higher number of NC treatments is required to achieve the same success rate as cIVF with a longer time to pregnancy [19–21].

In the present study, pregnancy rate per number of patients who get at least one transfer in COS group was higher being 28% compared with 18.85% of NC group.

Three hundred fifty-five conventionally stimulated patients achieved 845 embryos, and they needed 765 embryo transfers, while the 230 patients in the NC underwent 277 transfer with 277 embryos.

Nevertheless, analyzing the number of pregnancies per total of transfers and per total of embryos, the percentages were reversed.

In NC group, with less number of embryos, women get higher number of pregnancies, being the second most interesting finding the higher embryo implantation rate among patients who underwent NC rather than COS.

The transfer number in patients stimulated, indeed, was significantly higher but resulted in a lower number of pregnancies per transfer. We found 9.1% of pregnancies per transfer in stimulated women compared with 13% of pregnancies per transfer when embryos developed from natural follicles. This result reached statistical significance if the implantation rate was evaluated: the number of pregnancies for embryos transferred passed from 13% of the spontaneous cycle to 8.28% of cIVF.

Our results are particularly meaningful considering that embryo transfer procedure means for women to support an economic expenditure [21] of clinical and laboratory procedures and also an emotional expense related to the stress of undergoing an invasive procedure and to the hope of waiting for a positive pregnancy test.

Some studies hypothesized that implantation rate was lower in cIVF than in NC because of the dysregulation of endometrium for high estradiol concentration during conventional stimulation [19, 22], while other supported embryo quality as responsible [23–25]. The success of pregnancy rate of cIVF is directly correlated with the number of oocytes collected, so the chances are decreased in poor responders, while the success rate is higher when undergoing NC-IVF. A meta-analysis on conventional stimulation identified as negative prognostic factors for pregnancy: female age (OR 0.95, 95% CI: 0.94–0.96), duration of subfertility (OR 0.99, 95% CI: 0.98–1.0), basal FSH (OR 0.94, 95% CI: 0.88–1.0), and a positive correlation with oocytes number (OR 1.04, 95%CI:1.02–1.07) and embryo quality [26].

It must be also considered that emotional stress due to cIVF can cause premature treatment ending, thus reducing IVF success [27, 28]. Some contributing factors are daily injections, side effects, and costs but also embryo selection and cryopreservation [29].

This study has limitations of being retrospective and not homogeneous for the AMH levels, those were significant higher in stimulated patients compared with patients who underwent NC. Since AMH is a prognostic index of response to conventional ovarian stimulation [30], in the present study, patients with too low AMH did not receive pharmacologic stimulation as therapeutic solution but underwent natural cycle treatment. Therefore, two groups had different AMH values. However, although AMH of patients in natural cycle group was lower, they had a higher implantation rate. Furthermore, pregnancy rate per patients did not change between the two groups as a demonstration that AMH value did not correlate with pregnancy success, as recently described by von Wolff M et al. [31].

On the other hand, the strength points of this research were the large sample size and the homogeneity of the selected population in term of median age, BMI, FSH, menarche, previous pregnancies, AFC, and smoke status.

In conclusion our finding clearly showed that NC may be an option to consider in patients of advanced age identified as poor responder according to Bologna criteria, being outcomes comparable to cIVF but with a lower implantation failure.
